# A Cantonese Audio-Visual Emotional Speech (CAVES) dataset

**DOI:** 10.3758/s13428-023-02270-7

**Published:** 2023-11-28

**Authors:** Chee Seng Chong, Chris Davis, Jeesun Kim

**Affiliations:** The MARCS Institute for Brain, Behaviour and Development, Western Sydney University, Locked Bag 1797, Penrith, NSW 2751 Australia

**Keywords:** Cantonese dataset, Auditory and visual expressions, Emotional speech, Dataset evaluation

## Abstract

We present a Cantonese emotional speech dataset that is suitable for use in research investigating the auditory and visual expression of emotion in tonal languages. This unique dataset consists of auditory and visual recordings of ten native speakers of Cantonese uttering 50 sentences each in the six basic emotions plus neutral (angry, happy, sad, surprise, fear, and disgust). The visual recordings have a full HD resolution of 1920 × 1080 pixels and were recorded at 50 fps. The important features of the dataset are outlined along with the factors considered when compiling the dataset. A validation study of the recorded emotion expressions was conducted in which 15 native Cantonese perceivers completed a forced-choice emotion identification task. The variability of the speakers and the sentences was examined by testing the degree of concordance between the intended and the perceived emotion. We compared these results with those of other emotion perception and evaluation studies that have tested spoken emotions in languages other than Cantonese. The dataset is freely available for research purposes.

The study of emotional expression, both production and perception, is important for research areas interested in communication, knowledge representation, and culture. For example, understanding emotion expression and perception can provide a solid basis of research on human–human and human–machine interaction, how linguistic and paralinguistic information are simultaneously expressed, and whether and how social norms impact the production and perception of emotion. As such, progress in these research areas is underwritten by the development and availability of appropriate materials (corpora or datasets). The current work presents our work on a dataset of Cantonese audio-visual emotional speech (CAVES); below we present the background and aims of this endeavor.

A single dataset cannot serve the diverse interests of researchers who aim to study the expression of emotion. That is, the choices made in constructing a particular dataset predispose it to certain types of investigation. Consider how past studies have differed in what has been investigated and how these investigations were carried out. For example, many early studies of human emotion recognition were designed to maximize the control and standardization of experimental stimuli, and so often employed static facial expressions of emotions as conveyed by photographs (e.g., Ebner et al., 2010; Langner et al., 2010). This focus created a need for a standard set of expressions that would allow results to be compared across studies. Thus, rather than a dataset per se, standard sets of emotional face expressions were developed (e.g., the Pictures of Facial Affect, Ekman & Friesen, 1976).

In contrast, recent work has sought to compile rich emotion datasets that can consist of a very large number of multimodal (visual and auditory) instances depicting a broad range of emotions (e.g., Vidal et al., 2020). Typically, such datasets consist of extracts of talk shows, movies, interviews, real-life drama, etc., that have been selected based on various criteria which are then rated in terms of which emotion was expressed, or by a rating-by-comparison method (where two clips are presented and the one judged more positive selected, see Baveye et al., 2013). These stimuli better reflect real-life social experience; however, they can present numerous challenges with respect to measurement and comparison due to the large number of different talkers, differences in how the stimuli were collected (in recording quality, noise levels, etc.), differences in content, the variability of emotional displays, poses, framing and so on. As such, these datasets are better suited to machine learning applications rather than investigations that require controlled contrasts of specific variables.

In designing the current dataset, we adopted an approach intermediate between the two outlined above. That is, we made high-quality, consistent recordings of stimuli designed to allow a high degree of experimental control; but importantly, these stimuli captured the multimodal, i.e., auditory-visual dynamics of spoken emotional expressions. To get an idea of where the current dataset fits with others, Table 1 provides an overview of some selected auditory-visual speech emotion datasets. The table lists these datasets, the language of production, the number of speakers that contributed, the number of utterances recorded, the emotions expressed, how emotions were elicited and what access is available. As can be seen most of these datasets consist of spoken English, the number of speakers and utterances differ markedly, a core set of emotions were expressed, mostly acted, and typically the datasets are not publicly available.

**Table 1:** A comparison of key features of auditory-visual speech emotion datasets (2006–2021)

Dataset	Language	Speakers	Utterance	Emotion	Type	Access
CAVES	Cantonese	10	10425	A,D,F,H,N,S,Su	Acted	Yes
MHMC^1^	Mandarin	7	1680	A,H,N,S	Acted	No
NNIME^2^	Mandarin	44 (24 F)	3 min speech	A,D*,F*,H,N,S,Su	Acted	Contact
CHEAVD 1.0^3^	Mandarin	238 (113 F)	2322	A,D,H,N,S	Acted*	Not found
CHEAVD 2.0^4^	Mandarin	527 (219 F)	7030	A,A*D,H,N,O,S,W	Acted*	Not found
TUM AVIC^5^	English	21 (10 F)	3901	Nonverbal	Interaction	No
AFEW^6^	English	330	1426	A,D,F,H,N,S,Su	Natural	Contact
Fiction^7^	English	28 (12 F)	152 turns	F, negative, N,O	Acted*	No
RAVDESS^8^	English	24 (12 F)	7356 clips	A,C,D,F,H,N,S,Su	Acted	Yes
SEMAINE^9^	English	150	959	A,C*,D,F,H,S	Interaction	Request
SAVEE^10^	English	4 (M)	480	A,D,F,H,N,S,Su	Acted	No
eNTERFACE^11^	English	42 (8 F)	1186	A,D,F,H,N,S,Su	Acted	No
IEMOCAP^12^	English	10	3060	A,D,F,H,N,S,Su,O	Actors	Form
MELD^13^	English	6 (84%)	13000	A,D,F,J,N,S,Su	Acted*	Yes
SAFE^14^	English (80%)	400	4073	F, negative, N, positive	Acted	No
Kim & Davis^15^	English	5	10	A,D,F,H,N,S,Su	Acted	No
RECOLA^16^	French	46 (27 F)	4 min speech	5 Social Affects	Natural	Link down
Vera^17^	German	47	947	Valance, active, dom	Acted*	Link down
JAVED^18^	Japanese	14 (4 M)	100 min.	A, Content, H,N,S	Acted	No
HEU (part2)^19^	Multilingual	967	2435	A,B,D,D*,F,H,N,S,Su	Acted*	No
MDESVG^20^	Polish	16 (8 F)	560	A,D,F,H,N,S,Su	Acted	No
RAMAS^21^	Russian	10	581	A,D,H,S,S*,Su	Acted	Link down
BAUM-1^22^	Turkish	31 (13 F)	1222	A,D,F,H,S,Su,O	Interaction	Link down

Acted* = TV, movie, talk-show; A = anger; A* = anxious; B = bored; C = calm; C* = contempt; D = disgust; D* = disappointed; F = fear; F* = frustration; H = happy; J = joy; N = neural; S = sad; S* = scared; Su = surprise; O = other; W = worry

^1^Lin et al. (2012).

^2^Chou et al. (2017).

^3^Li et al. (2017).

^4^Li et al. (2018).

^5^Schuller et al. (2009).

^6^Dhall et al. (2012).

^7^Clavel et al. (2004).

^8^Livingstone et al. (2018).

^9^McKeown et al. (2011).

^10^Jackson & Haq (2015).

^11^Martin et al. (2006).

^12^Busso et al. (2008).

^13^Poria et al. (2018).

^14^Clavel et al. (2006).

^15^Kim & Davis (2012).

^16^Ringeval et al. (2013).

^17^Grimm et al. (2008).

^18^Lubis et al. (2016).

^19^Chen et al. (2021).

^20^Sapiński et al. (2018).

^21^Perepelkina et al. (2018).

^22^Zhalehpour et al. (2016).

The construction of the current CAVES dataset had several motivations, which can be summed up in terms of the dataset representativeness and research application. With respect to representativeness, the main aim of this work was to establish an emotional speech dataset for Cantonese. Cantonese is a major world language, and as far as we can tell no AV speech emotion datasets have been compiled or are available. Moreover, Cantonese is a tonal language (with two more phonetic tones than Mandarin), and somewhere between 40 and 60% of the world’s languages are classified as tonal languages (Maddieson, 2013; Yip, 2002), yet AV spoken emotion datasets almost exclusively use non-tonal languages (see Table 1).

In terms of research application, an important aim for CAVES was to provide a resource to examine a range of issues concerning how linguistic and emotional information are simultaneously expressed. More specifically, since spoken emotions and lexical tone is expressed via similar acoustic properties, e.g., fundamental frequency (F0) plays a key role in the production and perception of lexical tone and emotional prosody, the issue of how spoken tones affect the expression of emotion is a particularly interesting one. Studies (e.g., Anolli et al., 2008; Chong et al., 2015; Wang & Lee, 2015) have examined how variation in F0 is utilized to concurrently express linguistic and emotion information, but these studies were based on limited samples and emotions. A more extensive dataset would allow for a more comprehensive examination of the trade-offs between linguistic and emotional prosody for both auditory and visual expressions, e.g., the influence of dynamic versus static tones, the role of segmental and suprasegmental timing, whether the difference in F0 between female and male speech plays a role, and so on.

## CAVES versus other tonal language AV emotional speech datasets

As can be seen in Table 1, several other tonal language spoken emotion AV datasets have been developed. These, however, were established for a range of purposes different from the linguistic and psycholinguistic research application outlined above. In what follows, we identify some issues with these datasets; not intrinsic limitations per se, but rather limitations with respect to the current interest. To make these issues concrete, we focus on dataset scope and context (see Douglas-Cowie et al., 2003) and, from a practical viewpoint, on dataset availability.

The first dataset is the Multimedia Human–Machine Communication (MHMC) dataset (Lin et al., 2012). The language is most likely Taiwanese Mandarin although the language was not explicitly indicated. As far as we could determine, this dataset is not publicly available. The dataset used posed emotions produced by seven actors (both females and males, but no details of how many of each). Each actor said 30 sentences in four emotions (happiness, sadness, anger, neutral) and each utterance was repeated, thus there were 1680 utterances in total (2 × 30 × 4 × 30). The design context of the MHMC was that it was developed for automatic recognition of human emotions from audio-visual signals. The recordings were made in an office environment that had a reasonable level of foreground and background noise and video images were captured using a Logitech QuickCam at a low resolution of 320 × 240 pixels with a frame rate of 30 frames per second. The main issues with this dataset for current purposes are its lack of availability; the limited number of emotions captured, and the low quality of the AV recordings.

The second dataset is the National Tsing Hua University-National Taiwan University of Arts Chinese Interactive Emotion (NNIME) dataset (Chou et al., 2017). This dataset is not available online; and researchers must contact the first author to gain access. The design context was to capture dyadic human–human communication. This involved simultaneous recording of audio, video, and ECG signals collected during spoken interactions between 22 pairs of actors performing a spontaneous 3-min dialogue. The language is Taiwanese Mandarin. The emotions targeted for each performance were anger, sadness, happiness, frustration, surprise, and neutral. Emotion annotation was carried out by 49 annotators (the majority of which were peer reports from other actors). In all, 102 recording sessions were rated using a five-point scale on dimensions activation and valence. Video recordings were made via a fixed position camera that was positioned at a considerable distance from the actors who were free to move as within the field view of the camera. The main issues with this dataset are its lack of immediate availability; that the emotions captured do not cover the basic six emotions and were expressed using different utterances; and the low AV recording quality of facial expression due to the fixed distant camera, freely moving actors and profile views.

There are two other datasets, the CASIA Natural Emotional Audio–Visual Datasets (CHEAVD, Li et al., 2017) and CHEAVD 2.0 (Li et al., 2018) that appear to be available online for a fee (from the Chinese Linguistic Data Consortium), although how to arrange this was not apparent. The design context of these datasets was that they gather many speakers to enable speaker-independent emotion analysis. As such, the datasets were collected from media excerpts, e.g., for CHEAVD from 34 films, two television series, two television shows, one impromptu speech and one talk show (in Mandarin Chinese). Segments were selected based on clips that did not have high levels of background music or vocal overlap; the segment had only a single talker’s speech and face and contained a complete utterance. CHEAVD had 238 speakers (47.5% female) and CHEAVD 2.0 527 speakers (41.6% female). Segments were rated by four annotators that resulted in 26 emotional labels being used (neutral, angry, happy, sad, worried, anxious, disgust, surprise, blamed, sarcastic, aggrieved, curious, fearful, embarrassing, nervous, confused, proud, helpless, hesitant, contemptuous, frustrated, serious, anticipated, shy and guilty). The distribution of emotion labels was highly skewed (with some emotions having many and others with relatively few instances). It is unclear how the movie and television video clips were captured; however, videos were saved in 640 × 480 pixels with a frame rate of 25 fps, audio was saved at 44.1 kHz, stereo and 16 bit. The main issues with these two datasets are that they are not free for researchers; the emotions captured are unbalanced (i.e., few examples for some emotions); the emotions are expressed using different utterances and talkers; and the extent of background noise (both auditory and visual) was not specified.

CAVES contrasts with the above datasets. First, while there are some emotional speech datasets available for tonal languages, none exist for Cantonese. This is despite Cantonese being a language that is widely spoken and has a rich set of six phonetic tones. Also, compared to these datasets as well as most other ones in Table 1, the CAVES dataset is freely available for research purposes at https://forms.office.com/r/3VfeWQnAVa. For this style of dataset, the CAVES has a reasonably large number of talkers (*N* = 10), that had equal representation of female and male speakers. In addition, it has a large number of items (*N* = 50) that has good coverage of different tones at sentence initial and final positions (see below), and each sentence is expressed in each of the basic six emotions plus a neutral expression. Note that the six basic emotions were selected as they are considered to be universally expressed and recognized across cultures and are well studied and represented within the literature on cross-cultural research (Ekman, 1992). In all, then, there are 3500 AV stimuli and the same number of visual only and auditory only stimuli.

The context of the CAVES dataset design was for it to facilitate investigations of how linguistic and affective expressions interact, that is, how emotion and speech modulate facial expressions. To do this meant having controlled recording of talkers expressing the same lexical content in different emotions as this allows for differences in the expressive behaviors associated with each emotion to be readily contrasted. Having the same lexical content also controls for possible tone context effects and the influence of syntactic form on emotional expression (Cole, 2015). Moreover, having controlled talker recordings of predetermined content allowed a neutral semantic content to be used for all the emotions; this alleviates problems of perceivers being influenced by lexical content.

Below we present in more detail, a description of the design and development of the CAVES dataset (Part 1); and then report on a perceptual validation study (Part II). Note that this study was conducted in line with the principles of the Declaration of Helsinki. Approval was granted by the Ethics Committee of Western Sydney University (H10442).

## Part I: Dataset design and development

As mentioned above, the CAVES dataset contains six basic emotions (anger, disgust, fear, happy, sad, surprise) plus a neutral expression to serve as a baseline. We selected a set of semantically neutral carrier sentences so that all the six basic emotions could be expressed for each sentence without any semantic interference. Having a set of common carrier sentences allows the different emotions (and neutral baseline) to be straightforwardly compared. We also selected sentences that had a good coverage of the different lexical tones both in initial and final sentence positions. This selection allows for an examination of how the onset and offsets of emotion intonation change as a function of the different tones. It is not clear, for example, whether Cantonese speakers utilize different acoustic cues in expressing anger (associated with a rising tone) for sentences that have an onset/offset falling versus rising tones.

## Methods

### Participants

Ten native speakers of Cantonese (five females) who were born and raised in Hong Kong were invited to participate for monetary reimbursement. The average age of the participants was 29.1 years (SD = 4.9). All the participants also spoke English.

### Materials

Fifty sentences each containing ten syllables/characters were used. These sentences were selected from the 240 Cantonese Hearing In Noise Test (CHINT) sentence list^1^ (Wong & Soli, 2005). The selection was made on the basis that the sentences had a good distribution of different tones at initial and final positions. In selecting sentences in terms of the spread of the tones, a six-tone system was used (see Table 2).

**Table 2:**
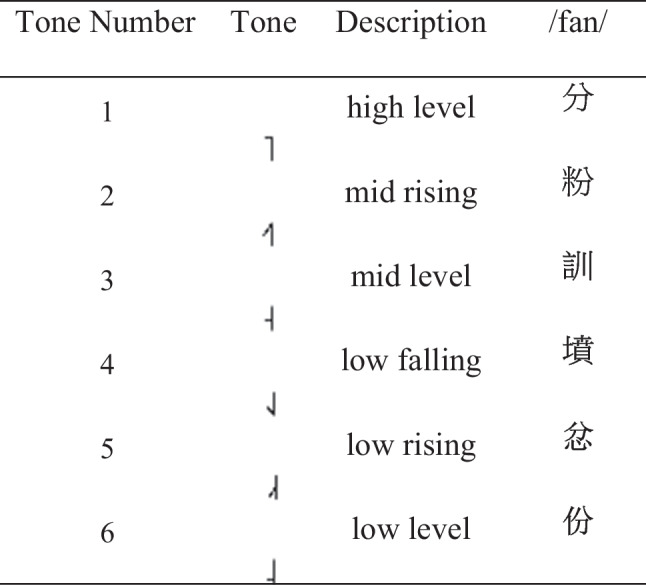
Example of the six tone classification system using the homophone /fan/

Here /fan/ is expressed in the six different tones and the English translation of these Chinese words from tone 1 to 6 are: “point”, “noodles”, “discipline”, “grave”, “angry” and “portion”

Table 3 shows the number of sentences with each tone in the initial and final positions for the 240 sentences in the CHINT and the 50 sentences that were selected to have a good balance of tones in the initial and final positions.

**Table 3:** Numbers of each tone in initial and final sentence position for the 50 items selected from the CHINT (the data for the full CHINT list are given for comparison)

Tones	240 original		50 selected	
	Initial	Final	Initial	Final
1	36	61	8	12
2	47	45	8	9
3	38	36	8	7
4	19	46	8	8
5	86	7	14	5
6	14	45	4	9

The numbers in each cell represent the number of sentences with the indicated tone in the initial and final position.

The CHINT sentences were developed to be used for hearing in noise tests, as such they included a list of parenthesized words that could be substituted and considered as a correct answer in a speech identification in noise paradigm. For example, in this sentence: 教授(就快 or 就嚟)去美國做研究^2^ both 就快 and 就嚟 have the same meaning, “soon”, so for the purposes of this dataset, we decided to use the second pair of characters 就嚟 to maximize the number of different tones within that sentence. To illustrate this, the original sentence represented in terms of tones would be: 3, 6, (6, 3 or 6, 4), 3, 5, 3, 6, 4, 3. So, to balance out the ratio of tone 3 to tone 4 characters we picked the second pair of characters so the sentence represented with these tones would be: 3, 6, 6, 4, 3,5, 3, 6, 4, 3. The same strategy was used for all the selected sentences to obtain emotional utterances that had a balance of all six tones. Including a balance of lexical tones will allow for the examination of how speech tones affect emotion expressions, e.g., how pitch changes in Cantonese especially on the different tones such as a high-level pitch contour in tone 2 or low/falling in tone 4 influence or are influenced by the expression of emotion.

### Recording setup

**Fig. 1:**
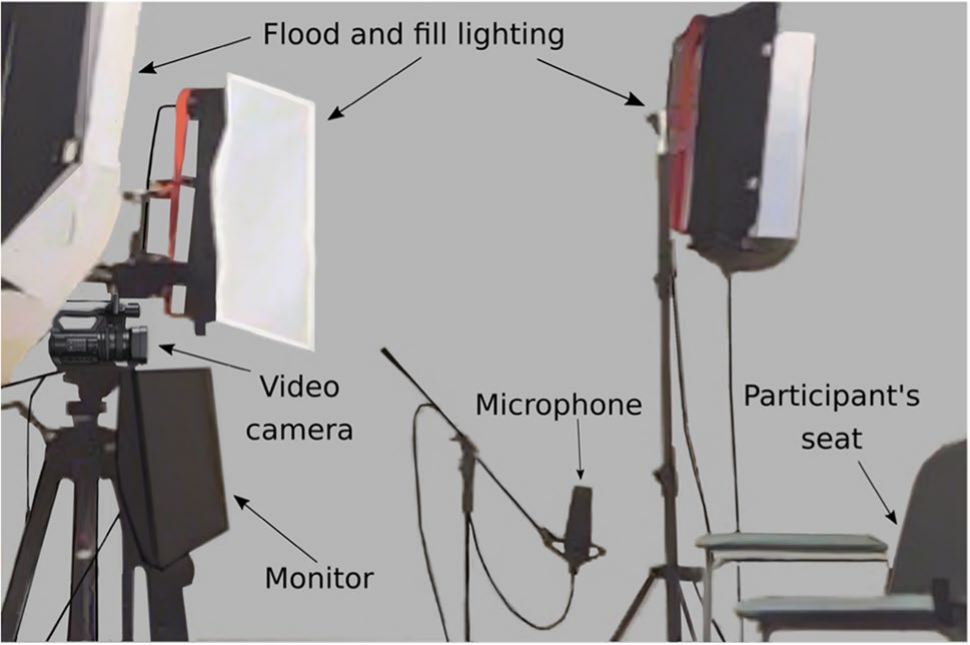
The setup in the recording booth showing the camera, screen microphone, lighting and participants’ seat

The recording was conducted in a sound attenuated booth at Western Sydney University. In the booth, participants were seated in front of a 20.1” LCD video monitor (Diamond Digital DV201B) that was used to present the stimulus sentences to the participant. Directly above the monitor was a video camera (Sony NXCAM HXR-NX30p) where participants were requested to fixate prior to uttering the sentences. The videos were recorded at 1920 × 1080 full HD resolution at 50 fps. To capture participants’ utterances a microphone (AT 4033a Transformerless Capacitor Studio Microphone) was placed about 20 cm away from the participants’ lips and out of the field of view of the camera (see Fig. 1 for a depiction of the setup). Audio captured using the microphone was fed into the Motu Ultralite mk3 audio interface with FireWire connection to a PC running CueMix FX digital mixer and then to Audacity which captured the sound at a sampling rate of 48 kHz. The camera, screen, and microphone heights were adjusted to suit each participant. The audio and video outputs were monitored by the experimenter who was seated outside of the booth.

All participants did a short trial session of three utterances to determine the best gain values for the microphones. Once an acceptable level had been achieved, the recording commenced.

### Procedure: Elicitation of emotion

Participants were briefed regarding the recording procedure before signing a written consent form indicating that recordings could be used for research purposes (the instructions and emotion labels were conveyed in English). Participants were instructed to be as natural as possible in how they expressed themselves and were asked to produce the emotions with the intent of communicating their emotional feelings to an observer. That is, rather than focusing on the inner experience of an emotion, the current interest was in the expression of emotional expressions for the purpose of communication, i.e., the emphasis was on the non-verbal signals people use to communicate emotion. As such, no emotional induction procedure was used and although the emotions were acted, the participants did strive to express each emotion as if she/he was conveying emotional information to another person. Table 1 shows that this type of emotion elicitation method is the most commonly used (see also Scherer, 2003).

The recording session was blocked by emotion type and the order of presentation was randomized across participants. In each block, participants were first informed of the emotion to express, and were then instructed to imagine themselves expressing this emotion to an interlocutor. They were then given three practice trials prior to the start of each block. Stimuli sentences were then displayed one at a time in a random order and the participants produced the utterances when ready. Participants were given feedback via the screen if they were required to repeat a sentence (e.g., if they misread the sentence or did not fixate on the camera while producing the expressions). They were given a break after the successful production of every 25 sentences. By the end of the recording session, all participants produced a total of 350 utterances (50 sentences × 7 emotional expressions including neutral).

### Data segmentation

The audio recordings were high-pass filtered (100 Hz) to remove noise and a noise-shaped dither was then applied. To reduce processing time, a down-sampled (16 kHz) copy of the audio recordings was used for segmentation purposes only. The Audio Segmentation Toolkit (Gravier et al., 2010) was used to automatically segment speech events from silence and the segmentations were exported as PRAAT textgrids (Boersma & Weenink, 2014). The segmentations were manually checked and corrected using MTRANS (Villegas et al., 2011). A buffer of 500 ms before and after the utterance was applied to capture articulatory and expressive gestures that may unfold before and after the utterance.

**Fig. 2:**
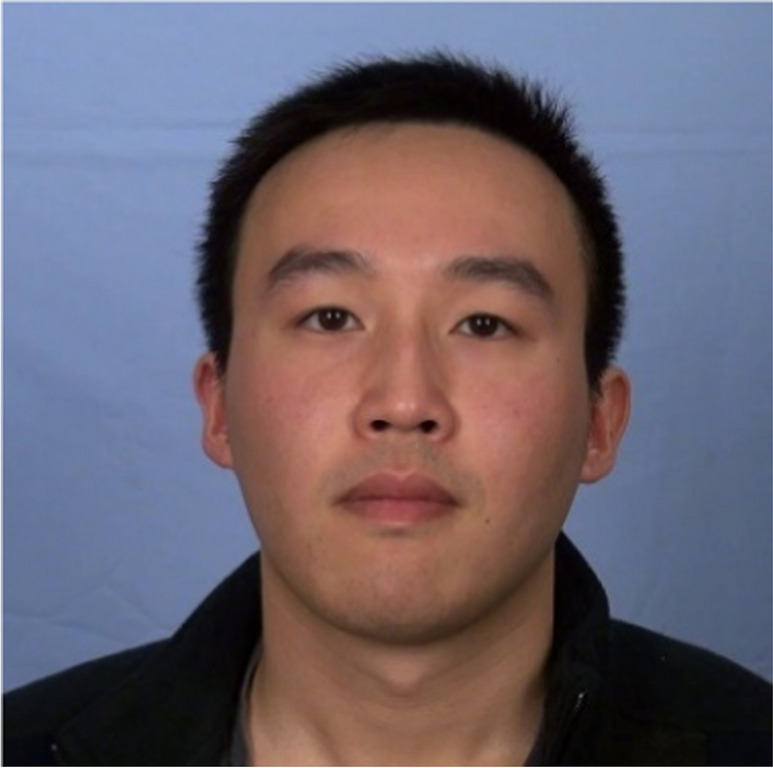
A single frame extracted from video clip to illustrate the extent to which the video was cropped

The corrected segmentations were used to extract individual sentence recordings from the uncompressed video (MTS, Advanced Video Coding High Definition format) and the audio recordings (48-kHz recordings). Audio-visual stimuli were then created by combining the video and audio recordings. The video files were cropped to a size of 1000 × 1000 pixels, framing the participants head within the center of the recording (see Fig. 2 for an example). We used a static cropping method which crops a predefined area (rather than a dynamic method which tracks and centers head movement). This method was used to capture the expressive head movements made by the participants to preserve rigid head motion which may carry emotion information (Davis & Kim, 2006; Kim et al., 2014).

All segmented video clips were kept in their original format (.MTS) to preserve the quality of the stimuli. These clips (10 speakers × 7 (6 basic emotions + neutral) × 50 sentences = 3500) were labeled by speaker ID, emotion type and then by sentence ID, except 25 sentences produced by one female speaker in the Sad condition that were lost due to technical issues.

### Part II: Validation of the CAVES dataset

The recorded expressions from the CAVES dataset were evaluated using an emotion identification experiment. The primary aim of this experiment was to assess the validity of the produced spoken emotion in terms of the degree of concordance between the perceived emotion expression and the intended emotion expressed by the speaker. For this, we examined how emotion recognition accuracy rates and the distribution of response frequencies from each spoken expression varied as a function of emotion type and presentation condition. The results were also compared to the findings of other emotion perception and evaluation studies particularly using spoken emotions in languages other than Cantonese.

The secondary objective of this experiment was to determine the reliability of the dataset by examining how variable speakers and sentences were in terms of accuracy rates (for speakers and sentences). Results from this examination can be used as a reference for the selection of stimuli in future studies.

## Methods

### Participants

Fifteen participants (including ten females, mean age = 23.2 years, SD = 3.9) took part in this study for a small payment. All participants were native listeners of Cantonese who were born and raised in Hong Kong. The participants spoke both English and Mandarin*.* The majority of the participants were recruited through word of mouth and snowball sampling procedures. This sample size was selected based on it being a feasible sample size to run given how long each participant took to complete the experiment and the availability of Cantonese speakers. This sample size yielded effects sizes for each of the main contrasts (i.e., AV vs. VO; AV vs. AO and VO vs. AO) for each emotion that ranged from small to medium (Cohen, 1988) (see Table 3). Effect sizes were calculated based on the methodology published in Westfall et al. (2014).

### Stimuli

All speech recordings (50 sentences × 7 (6 emotions and neutral) expressions × 10 speakers including five females) were used as stimuli, except 25 sentences by one female speaker in the Sad condition. These recordings were presented in three presentation conditions, auditory-visual (AV), visual-only (VO) and auditory-only (AO), resulting in a total of 10,425 stimulus items.

### Design and procedure

Due to the large number of stimuli, each participant was tested over multiple sessions. Each session was conducted on a separate day and consisted of a total of 900 trials (50 sentences × 3 presentation conditions × 6 emotions, note: the neutral expression was used as a speaker-specific baseline, see below) from a random selection of either male or female only speakers.

Although we aimed to conduct a fully within-subjects design by having each participant rate all the speech recordings, not all participants were able to fully complete the validation study due to limits on availability (judging the large number of recordings in this dataset required a considerable time commitment). Nevertheless, participants were encouraged to participate in as many sessions as possible, up to the maximum of ten (where all recordings would be tested). As an eligibility criterion to participant in the experiment, all participants agreed to participate in at least five sessions of the study. In all, 51,408 judgments were obtained from participants. The number of these judgments were split evenly across emotion type (17% each), presentation type (AV = 31%; VO = 37%; AO = 32%) and female/male (38 and 62 %) and individual talker (8–13%).

The stimuli were presented using DMDX (Forster & Forster, 2003) on a 15.6-inch laptop (Lenovo T520) that is connected to an EDIROL UA-25ex soundcard with Sennheiser HD550 headsets. Participants were tested individually in sound-attenuated IAC booths at Western Sydney University.

Participants were given written instructions and a short practice session prior to the start of the experiment. In the practice session, participants were first presented with two video clips of the speaker uttering a sentence in a neutral expression. These neutral expressions were included to help familiarize the participants with the speaker and acted as a speaker specific baseline against which to judge the emotional expressions. The neutral utterances were followed by 12 practice trials. Each trial consisted of one emotion expression and participants were required to identify the emotion by responding to a six alternative forced choice task using the mouse. Note, these practice trials were presented later as experimental trials to be again rated by the participants. The researcher remained with the participant during the practice session to ensure that the participants understood the task.

The experimental trials were presented in the same format as the practice trials. The trials were blocked by presentation condition for each speaker. Participants were always given two sentences in a neutral expression at the beginning of each block. The presentation order of the blocks was counterbalanced across the speakers. With no time limit imposed, participants could proceed at a pace that they were comfortable with. Participants were given a 5-min break every 150 trials and reimbursed for their time at the end of each session.

### Analysis

The first set of analyses was conducted on the participants’ accuracy data in recognizing the different types of emotions across the three presentation conditions (the analysis was also conducted using unbiased Hu scores, Wagner, 1993). Using the findings of other studies as a benchmark, it was expected that (1) bimodal emotion expressions (AV) be recognized with higher accuracy than the unimodal expressions of VO and AO (see Kim & Davis, 2012); and (2) recognition accuracy would vary as a function of emotion type, e.g., expressions of Happy were expected to be recognized with the highest accuracy while expressions of Fear at the lowest accuracy (Ebner et al., 2010; Langner et al., 2010; Tanaka et al., 2015; also see Scherer, Banse & Wallbott, 2001 for auditory only expressions). The rest of the analyses consist of providing descriptive statistics, examining confusion matrices, and exploring speaker and item level differences in accuracy scores. The perception data is available at https://forms.office.com/r/3VfeWQnAVa.

## Results and discussion

**Accuracy**

**Fig. 3:**
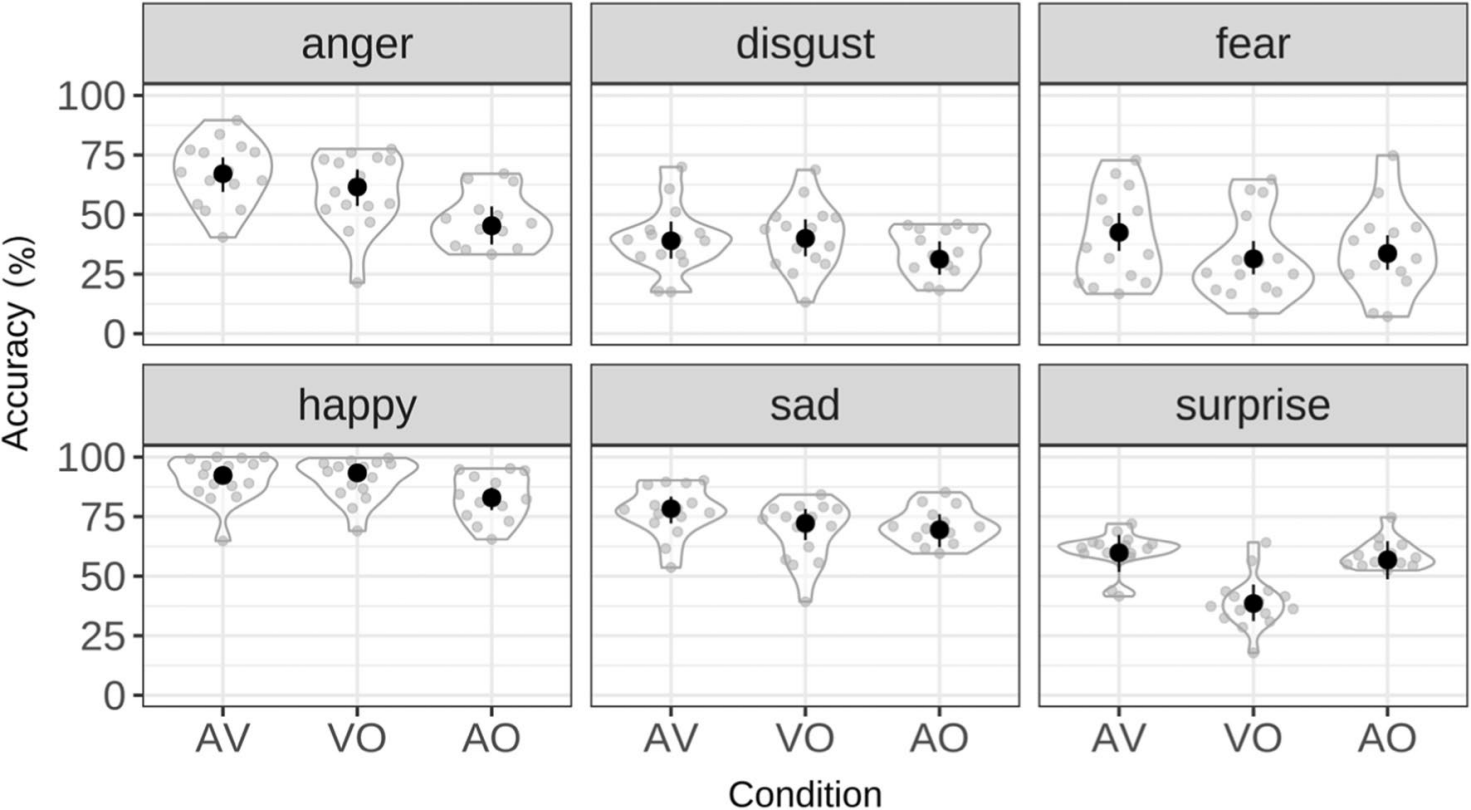
Percent accuracy scores for all emotion types by presentation conditions (model-based standard error)

Figure 3 presents the recognition accuracy scores across the six emotion types for each presentation condition. A generalized linear mixed model fit by maximum likelihood (Laplace Approximation) [glmerMod], Family: binomial (logit) was fitted to the data to examine if recognition accuracy varied as a function of presentation condition, emotion type and the interaction between the two. The R afex package (Singmann et al., 2021) was used to build the model and the ggplot package (Wickham, 2016) and afex_plot package (Singmann et al., 2021) was used to generate all the graphs presented in this paper. Speaker, participants, and sentence were entered as random factors, emotion type and presentation condition as fixed factors, and recognition accuracy as the dependent variable (formula: mixed(Accuracy ~ Emotion type * Presentation condition + (1|Participant) + (1|Item) +(1|Speaker), data = CAVES_data, family = binomial, method = "LRT", all_fit = TRUE)). Note that attempting to generate maximal or near maximal models (e.g., add in random slopes to any of the random variables) led to failures to converge, thus we accepted a simpler model, rather than risk the problems associated with fitting overparameterized models (see Matuschek et al., 2017).

Both the main effects and the interaction between them were significant; emotion type, χ2(5) = 7714.38, *p* < .001; Presentation condition, χ2(2) = 327.77, *p* < .001; and the interaction between these variables, χ2(10) = 600.16, *p* < .001. One-sample *t* tests with Bonferroni correction indicate that expressions of all emotion types were recognized at above chance accuracy (16.7% = 100% divided by six possible response options) across all presentation conditions.

As mentioned above, the accuracy scores were also converted to unbiased Hu scores (Wagner, 1993); since these are proportions these scores were arcsine transformed. The transformed Hu scores were then analyzed with a within measures repeated ANOVA (using the AFEX package, formula: Hu score (arcsine) ~ Emotion type*Presentation condition + Error(participant/Emotion type*Presentation condition). The outcome of the analysis agreed with that of the unadjusted recognitions scores, Emotion type *F*(2.24, 26.92) = 55.28, *p* < .001, Partial Eta squared = .822; Presentation condition, *F*(1.91, 22.96) = 16.97, *p* < .001, Partial Eta squared = .586; and the interaction between Emotion type and Presentation condition, *F*(4.39, 52.7) = 16.25, *p* < .001, Partial Eta squared = .575.

Further statistical significance testing was conducted using the emmeans package (Lenth, 2021) and the results are shown in Table 4. *P* values were adjusted using the Tukey method for comparing a family of three estimates. These tests were conduction on the recognition scores and the (arcsine transformed) Hu scores. As can be seen in the table, the pattern of the outcome of the analysis was the same for the simple recognition scores and the unbiased (transformed) Hu scores.

**Table 4:** Effect size (d) and *p* values of the pairwise contrasts between presentation condition and emotion type for the simple recognition (*middle*) and bias-corrected Hu scores (*bottom row*)

Emotion	AV vs. VO	AV vs. AO	VO vs. AO
Anger	d = 0.05 Z = 4.4, *p* < .001	d = 0.20 Z = 16.5, *p* < .001	d = 0.15 Z = 13.1, *p* < .001
	t = 6.54, *p* < .0001	t = 11.28, *p* < .0001	t = 4.60, *p* < .001
Disgust	d < 0.01 Z = – 0.70, *n.s.*	d = 0.08 Z = 6.2, *p* < .001	d = 0.08 Z = 7.4, *p* < .001
	t = 0.30, *p* = .77, n.s	t = 3.42, *p* < .006	t = 2.89, *p* < .05
Fear	d = 0.11 Z = 9.2, *p* < .001	d = 0.08 Z = 7.0, *p* < .001	d = 0.02 Z = – 2.0, *n.s*
	t = 3.47, *p* < .0046	t = 2.73, *p* < .05	t = – 0.71, *p* = .49, n.s
Happy	d = 0.01 Z = – 1.1, *n.s.*	d = 0.09 Z = 7.6, *p* < .001	d = 0.11 Z = 9.3, *p* < .001
	t = – 0.42, *p* = .68, n.s	t = 3.75, *p* < .01	t = 9.29, *p* < .0001
Sad	d = 0.06 Z = 4.9, *p* < .001	d = 0.08 Z = 6.7, *p* < .001	d = 0.02 Z = 2.1*, n.s.*
	t = 3.75, *p* < .003	t = 2.63, *p* < .05	t = – 1.88, *p* = .08, n.s
Surprise	d = 0.20 Z = 17.0, *p* < .001	d = 0.03 Z = 2.1, *n.s.*	d = 0.18 Z = – 15.3, *p* < .001
	t = 5.50, *p* < .0001	t = 0.01, *p* = .99, n.s	t = – 3.77, *p* < .01

In general, the patterns in accuracy rates observed in this study were similar to those of other AV speech studies, e.g., Kim and Davis (2012) that examined spoken expressions of English presented in the three different presentation conditions. Accuracy in the AV condition was significantly higher than both VO and AO conditions for all emotion types except for Disgust and Happy; VO was as accurate as AV for these two emotion types. Comparing the VO to AO condition, accuracy was significantly higher in the VO condition for Anger, Disgust and Happy. This result is also closely aligned with the findings of Kim and Davis (2012) that Anger, Disgust and Surprise were recognized more accurately in the VO compared to AO condition. In the current results, Surprise was the only emotion type where accuracy in the AO condition was significantly higher than the VO condition.

Collapsing across presentation modalities, Happy was recognized at significantly higher accuracy rates than other emotion expression (*p* < .001). Disgust and Fear were recognized at significantly lower accuracy than all other expressions (*p* < .001). A similar pattern of emotion recognition was reported in the evaluation of the Faces, Radboud and Karolinska Directed Emotional Faces dataset (Ebner et al., 2010; Langner et al., 2010; Goeleven et al., 2008). The finding that spoken expressions of Fear were recognized with the lowest accuracy was similar to that observed in the Tanaka et al. (2015) study which examined spoken expressions of emotions produced by Japanese and Dutch speakers.

### Confusion matrices

Tables 5–7 show the confusion matrices for the three presentation conditions. Expressions of Anger were either misidentified as Disgust (AV and AO) or Sad (VO). Expressions of Disgust were either misidentified as Anger (AO) or Sad (AV and VO). Confusion between Anger and Disgust is a common finding observed in evaluations of facial and spoken expressions (Kim & Davis, 2012; Tanaka et al., 2015). It was further observed that negative emotions such as Anger, Disgust and Fear were typically misidentified as Sad; a finding that aligns with previous evaluations of static facial expressions which found Sad to be the most frequently selected response (Goeleven et al., 2008).

**Table 5:** Confusion matrix for the AV condition

				Response			
		Anger	Disgust	Fear	Happy	Sad	Surprise
Presented emotion	Anger	**69.1**	10.2	3.4	8.5	7.3	1.5
	Disgust	6.4	**41.6**	17.4	5.6	18.4	10.7
	Fear	4.2	11	**43.9**	8	26	7
	Happy	0.4	0.4	0.8	**91.5**	1.7	5.1
	Sad	2.4	6.5	4.8	7.8	**78**	0.4
	Surprise	1.4	1.7	5.7	27.7	2.5	**61**

For Tables 5, 6 and 7, percent correct emotion identification is in bold.

**Table 6:** Confusion matrix for the VO condition

				Response			
		Anger	Disgust	Fear	Happy	Sad	Surprise
Presented emotion	Anger	**62.3**	8.2	5.2	7.2	14.9	2.2
	Disgust	7.8	**42**	19.3	5.2	21.5	4.1
	Fear	9.1	13.9	**33.7**	8.1	27.5	7.6
	Happy	0.6	1.1	0.9	**92.8**	3.5	1.1
	Sad	7.6	7.7	6	5.6	**72.1**	0.9
	Surprise	7.5	4.7	5.8	34.6	6.7	**40.6**

**Table 7:** Confusion matrix for the AO condition

				Response			
		Anger	Disgust	Fear	Happy	Sad	Surprise
Presented emotion	Anger	**48.7**	29.7	2.4	10.6	5.8	2.9
	Disgust	22.6	**35**	10.9	7.1	13.4	11
	Fear	4.9	6.6	**37.4**	14.7	30.3	6.1
	Happy	2	4.8	1.9	**83.6**	6.3	1.4
	Sad	0.9	6.9	7.2	12.9	**71.1**	1.1
	Surprise	6.4	2.8	4.2	23.9	3.3	**59**

Fear was misidentified as Sad across all presentation modalities. This was similar to the results reported by Tanaka et al. (2015) and by Banse and Scherer (2001). This is, however, in contrast to some studies that have reported that Surprise is the most likely alternative response (see Goeleven et al., 2008; Biehl et al., 1997). Expressions of Happy and Fear were rarely confused with other emotion types.

**Fig. 4:**
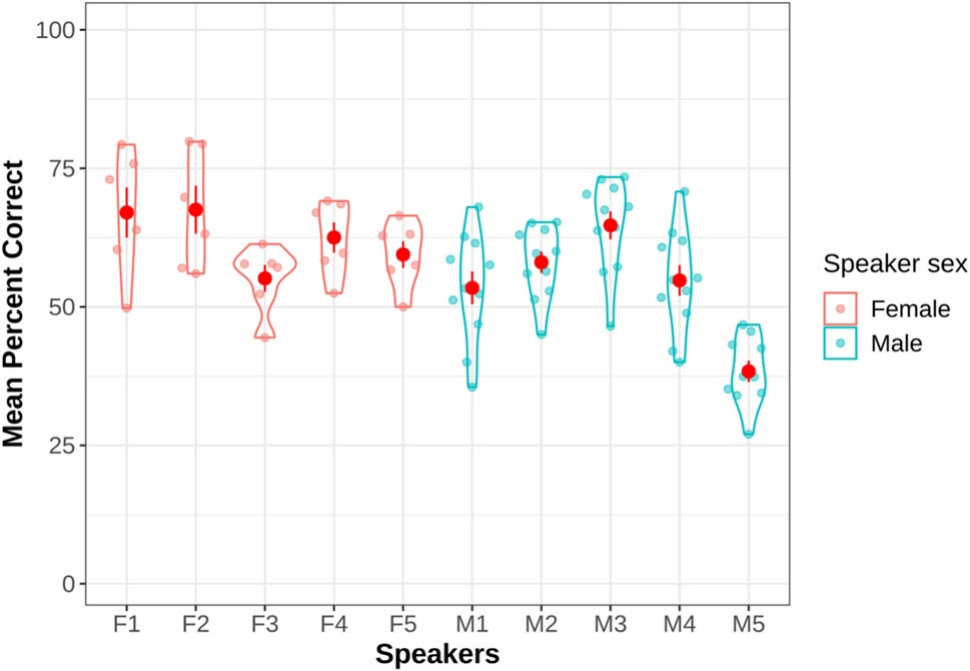
Mean percent correct recognition score for each speaker in the CAVES dataset. *Note.* Female speakers were given identifiers that started with ‘F’ with a number from 1 to 5 to denote each individual speaker. Similarly, males were given identifiers that started with ‘M’

Interestingly, expressions of Sad in the AO condition were at times misidentified as Happy, further investigation of the data suggests that this was mainly driven by the stimuli produced by one of the male speakers (M5 whose expressions were recognized with the lowest accuracy scores, see Fig. 4). Across all presentation conditions, expressions of Surprise were most likely to be misidentified as Happy which is also a commonly observed finding (see Kim & Davis, 2012; Tanaka et al., 2015).

### Variability of speakers

Figure 4 shows participants’ mean percent accuracy score for identifying emotion expressions that were produced by each of the ten speakers in the CAVES dataset. The variability is reasonably low, i.e., there were high agreement across the perceivers in identifying the speakers’ emotions, indicating the judgments and the expressions were reliable (Biehl et al., 1997). We see also calculated Gwet’s AC1 inter-rater reliability scores (Gwet, 2014) using the irrCAC R package, version 1.3 (Gwet, 2023). This was done using the recognition scores as a measure of inter-rater reliability, i.e., by assessing how often a presented emotion was recognized as that emotion (the correct count data). Given that the interpretation of AC1 scores is like the kappa statistic, AC1 ranges from almost perfect disagreement (– 1.00) to almost perfect agreement (+ 1.00) and a score of zero indicates chance reliability, we can use the nomenclature of Landis and Koch (1977). Based on this, the CAVES emotion reliability ranged from slight (0.00–0.20) to almost perfect (0.81–1.00), with most falling in the fair (0.21–0.40) to moderate range (0.41–0.60), i.e., AC1 disgust = 0.15 (SE = .030); AC1 fear = 0.2 (SE = .037); AC1 = 0.393 SE = .023); AC1 anger = 0.45 (SE = .048); AC1 sad = 0.6 (SE = .041); AC1 happy = 0.84 (SE = .039).

A Kruskal–Wallis test indicated that emotion expressions produced by female speakers were recognized at a higher accuracy than male speakers χ2(1) = 318.5, *p* < .001. This is a common finding in the literature of emotion perception studies (for example, see Wells, Gillespie & Rotshtein, 2016).

Tukey HSD tests indicated that expressions produced by speaker F2 and F1 were recognized at significantly higher accuracy rates than all other participants (*p* < .001) except M3. The difference between F1 and F2 was not significant. Accuracy at recognizing the expressions produced with speaker M5 was significantly lower than all the other speakers (*p* < .001).

### Variability of sentences

**Fig. 5:**
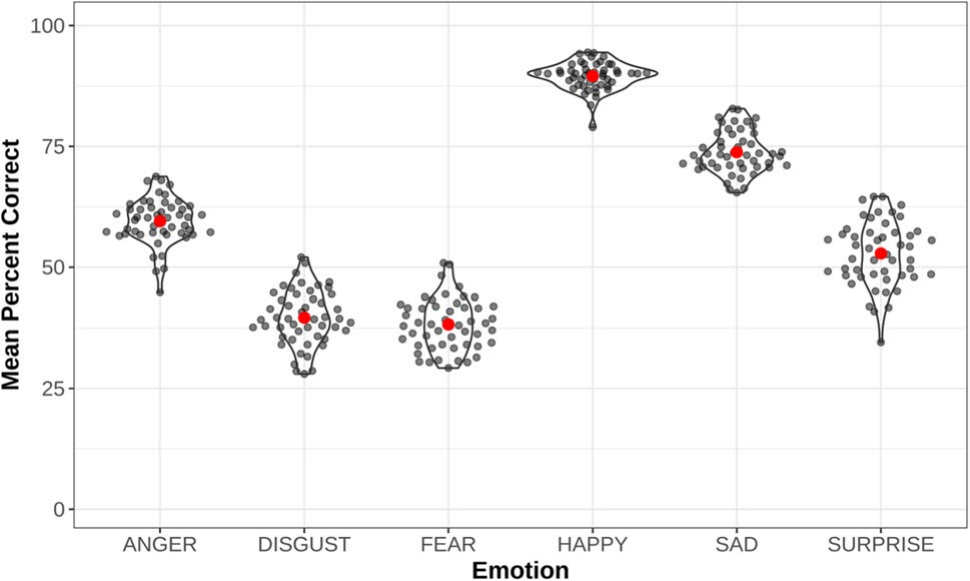
Mean percent correct recognition scores for all 50 sentences across the six emotion types

There were a total of 50 different sentences that were recorded in the CAVES dataset. Collapsing across emotion type and speakers, participants recognized all sentence stimuli within the range of 54–63%. For 39 out of 50 sentences, the emotion recognition accuracy rates were within a range of 56 to 59%. Figure 5 shows the distribution of scores for all sentences for each emotion type and the outliers (i.e., sentences with identifiers of 0410 and 0510).

Overall, the variability in accuracy scores across individual sentences is relatively low, indicating that these sentences are similar in their characteristics relevant to emotion expressions by Cantonese speakers, thus confirming the validity of our emotion selection and manipulation procedure (see Table 2). Further, the low variability suggests that the selected sentences (with the exception of a few outliers) which were semantically neutral did not bias responses to any particular emotion type.

In sum, the emotion expressions of the CAVES dataset can be recognized at above chance accuracy rates; show a significant AV benefit effect, and the response distribution frequencies are more or less similar to those of other emotion perception and evaluation studies (e.g., Kim & Davis, 2012; Li et al., 2017) but do differ from the RAVDESS (Livingstone & Russo, 2018). While differences with other studies were found in the patterning of the confusion matrices, these differences were likely driven in part by idiosyncratic individual differences (speaker M5 for example). Cultural or language differences between Cantonese speakers and the other language speakers in the other studies may also have contributed to different outcomes (e.g., English speakers in Kim & Davis 2012; Japanese and Dutch in Tanaka et al., 2015; and German expressers of the Radboud dataset, Ebner et al., 2010).

It is also worth pointing out a caveat related to the evaluation study. As mentioned above, due to its size, it was impractical to have all participants assess all items in the dataset. Thus, although there were approximately equal number of stimulus judgments made at the level of emotion and presentation type there were unequal numbers of judgments per item. This approach to evaluation could introduce variability in participant judgments for different items that may have affected the overall findings.

## Discussion and potential applications

We have developed a dataset of auditory and visual expressive speech in Cantonese (CAVES). This dataset provides a new research tool for studying spoken emotional expression in a tonal language (Cantonese). The dataset consists of 3000 high-quality Audio-visual recordings by ten (five female) native Cantonese speakers. Items were validated by 15 native Cantonese perceivers. Overall, each emotion was recognized at accuracy levels greater than chance (Anger = 60.3%, Disgust = 39.5%, Fear = 38.3%, Happy = 89.3, Sad= 73.7, Surprise = 53.3, chance = 16.7%). The expression ‘happy’ was the most accurate for all presentation modes (AV, VO, and AO).

The dataset provides a useful resource for research on such topics as the auditory and visual expressive speech (see Kim, Bailey & Davis, 2018), on how emotions are expressed in a tonal language, and on how emotions are expressed in speech. For example, in the auditory domain, the background-noise free, high quality auditory stimuli allow the precise measurement of the acoustic parameters associated with tones (e.g., F0-based measures, time-based measures) that can be contrasted across emotions and compared to the neutral condition (here, the ProsodyPro suite of Praat scripts is an excellent measurement resource, Xu, 2013).

The CAVES dataset provides an excellent resource to systematically explore how emotional speech is expressed in a tonal language for both auditory, visual, and auditory-visual presentation. In general, it has been shown that when vocal emotions are expressed in a tonal language, a restriction in pitch variation occurs compared to non-expressive speech (Ross et al., 1986; Anolli et al., 2008; Chong et al., 2015). More recent work in Mandarin (that has four phonetic tones, tone 1 - high level; tone 2 - rising; tone 3 - low/dipping, and tone 4 - falling), has explored this interaction between the expression of lexical tone and emotion in more detail. For example, in a series of studies that examined the auditory expression of emotional speech in Mandarin, Wang and colleagues found that variation in F0 in emotion expression was reduced for tone 1 (Wang & Lee, 2015; Wang & Quian, 2018; Wang et al., 2018). That is, Wang and Lee (2015) examined the expression of happy, neutral, and sad and found that a restriction in F0 variation occurred in all high, level tone sequences (tone 1 group) for the expression of happiness but did not happen for the dynamic tones. Wang and Quian (2018) replicated Wang and Lee (2015) but added in two more emotions (anger and fear). The results confirmed that the expression of different emotions had a restricted F0 range for tone 1.

These studies also showed that even though tone 1 had restricted pitch variation, the expressed emotions were well recognized. To explain this, it was proposed that speakers and listeners used other cues (e.g., duration, pitch and intensity) to express and identify emotions, i.e., that when a certain cue (e.g., pitch) is restricted in one language, other cues will be exaggerated to allow the vocal emotions to be identified (Wang et al., 2018). To determine whether emotion expression or the expression of tones is affected more by their interaction, Chang et al. (2023) conducted a perception experiment in Mandarin for angry, fear, happy, sad, and neutral expressions. Listeners were asked to identify the tones or the emotion expressed and the results showed that emotions affect Mandarin tone identification to a greater extent than Mandarin tones affect emotion recognition.

In addition to examining how lexical tone affects the perception of emotion and vice-versa, the Chang et al. (2023) study also conducted acoustic analyses of F0 (mean and range),mean amplitude and duration. Although they found that emotional expression influences Mandarin tone production and did so to different degrees depending on which Mandarin tones were spoken and which emotions expressed, the pattern of their results was at odds with earlier work showing a restriction F0 range for tone 1 (e.g., for tone 1, anger had a larger F0 range than the emotion neutral baseline). Chang et al. suggested that differences in methodology and materials may have led to the discrepancy between their results and others.

In our view, the CAVES dataset offers a unique resource for further probing and understanding how the expression of lexical tone and emotion type interact. For example, the CAVES dataset, with its large number of instances, allows for a machine learning approach to the issue of how lexical tone and emotional expressive speech may interact in the richer tone space of Cantonese. Recently Kanwal et al. (2022) have developed an auditory emotion classification method using robust features that achieved state of the art correct emotion classification from the English language RAVDESS emotion dataset (Burkhardt et al., 2005) and the German language EMO-DB emotion dataset (Livingstone & Russo, 2018). Applying this method to the CAVES dataset, and comparing the overall correct classification rates with those of the RAVDESS and EMO-DB, as well as comparing the confusion matrices, will reveal whether and how the expression of lexical tone affects emotion classification.

Likewise, in the visual domain, the high-quality videos allow for markerless tracking of head and face motion (e.g., using the openface software package, Baltrusaitis et al., 2018). A comparison of emotion classification based on the visual properties of emotion across tone and non- tonal language datasets is interesting given claims that the expression of lexical tone affects head and face motion (Burnham et al., 2022). In addition to using classification models, the relative performance of human perceivers (for both auditory only and visual only presentation) on these datasets would also be an option.

Speech in noise (emotion in noise) recognition is also a research area that will benefit from the CAVES dataset. Recent studies have suggested that emotional speech is more intelligible in noise than neutral speech (e.g., Gordon & Ancheta, 2017; cf., Davis et al., 2017). The CAVES dataset provides an opportunity to investigate the basis of this effect for a larger set of emotions and talkers. The CAVES dataset also provides the materials to investigate the perception of lexical tones in noise. This type of investigation is important for assessing design choice for the language model of cochlear implants for tone speakers (see Wong et al., 2018). Not only does the CAVES dataset allow for comparison of different tones and tone position to be tested, it also enables any effect of emotional expression and visual speech to be determined.

Another research area for which the CAVES dataset will prove useful concerns the factors that modulate emotion recognition performance, i.e., why some depictions of spoken emotion are better recognized than others. That is, it has been proposed that stimuli from talkers who produced more consistent emotion portrayals will be better recognized (see Davis & Kim, 2019). Here, the high quality auditory and visual stimuli of the CAVES dataset allow within and across talker consistency of auditory and visual properties to be easily assessed.

## Footnotes

1 The CHINT was used under licence from the University of Hong Kong; and House, Ear Institute, which restricts the full listing of the sentences used. In the CHINT, sentence-final particles (SFP) were avoided. The list of 50 sentences (no SFP) is available from the first author on reasonable request.

2 In English, this sentence reads “The professor will soon be travelling to America to conduct research”.
