# Author Correction: A Cantonese Audio-Visual Emotional Speech (CAVES) dataset

**DOI:** 10.3758/s13428-023-02318-8

**Published:** 2023-12-11

**Authors:** Chee Seng Chong, Chris Davis, Jeesun Kim

**Affiliations:** The MARCS Institute for Brain, Behaviour and Development, Western Sydney University, Locked Bag 1797, Penrith, NSW 2751 Australia

**Author Correction: Behavior Research Methods**

10.3758/s13428-023-02270-7

The original online version of this article was revised: the authors mistakenly left author Julián Villegas off the reference for M. Cooke. The reference has been updated.

